# Melting Behavior and Phase Transition Characteristics of Superalloy FGH96 Powder and Bulk Material During Vacuum Induction Melting

**DOI:** 10.3390/ma19143059

**Published:** 2026-07-16

**Authors:** Wei Sun, Runfang Xiang, Fuyang Cao, Lunyong Zhang, Jianfei Sun, Yongjiang Huang

**Affiliations:** 1 National Key Laboratory for Precision Hot Forming, Harbin Institute of Technology, Harbin 150001, China; 2 School of Materials Science and Engineering, Harbin Institute of Technology, Harbin 150001, China

**Keywords:** vacuum induction melting, multiphysics modeling, recycling, Ni-based superalloy scraps

## Abstract

Vacuum induction melting (VIM) of recycled powder with bulk master alloy represents an industrialized approach for recycling metallic waste. However, the intrinsic mechanisms governing the co-melting behavior of materials with distinct melting characteristics, such as powder bed and bulk alloy, remain insufficiently understood. To address this, a coupled multiphysics model was developed to simulate the evolution of induction melting involving homogeneous alloys with different morphologies. This model integrates magnetic, electric, and phase-field dynamics while incorporating melt convective heat transfer, thereby establishing a fully coupled electromagnetic-thermo-hydrodynamic framework. Through this modeling approach, the entire VIM process of melting homogeneous alloy with different morphologies can be comprehensively analyzed. The validity of the model was verified via small-scale VIM experiments using FGH96 powder/bulk composite, supported by infrared temperature measurements. This simulation methodology is not only applicable to small-scale recycling but can also be extended to large-scale industrial production, providing a reliable theoretical foundation for the recycling of powder materials.

## 1. Introduction

Powder metallurgy superalloys, such as FGH96, are extensively utilized in the manufacturing of turbine disks for advanced commercial aero-engines due to their exceptional properties, including high strength and thermal resistance [1,2]. However, during the gas atomization process, substantial quantities of off-spec powder are generated, with only 30% to 50% of the powder falling within the target particle size range suitable for powder metallurgy applications [3,4]. This results in significant material waste and low utilization rates, ultimately contributing to the high overall cost of aero-engines. Therefore, the recycling of off-spec powder has become a key solution for reducing the manufacturing costs of commercial aero-engines.

Current research on the recycling of off-spec powder has primarily focused on processes within powder metallurgy [5,6,7], while the re-manufacturing process of such powder remains largely unexplored in the literature. Recycling this off-spec powder through VIM in combination with master alloys presents a novel and efficient approach for remelting and re-atomizing the material. This technology ensures compositional uniformity and precise temperature control, effectively enhancing material recovery and heating efficiency. It offers a promising pathway for improving powder utilization and reducing waste, addressing critical technical challenges in large-scale powder recycling.

Compared with the melting process of bulk materials, the VIM-based recycling of off-spec powder with master alloys differs significantly due to the non-uniform melting characteristics across regions of the homogeneous material with heterogeneous properties. This leads to notable differences from conventional melting behavior. However, this issue, which is crucial for powder recycling, has received little attention. In recent years, advanced multiphysics simulation techniques have been widely employed to study the melting behavior of superalloys, enabling accurate predictions of temperature and flow field distributions. Among various simulation methods, the Finite Element Method (FEM) is regarded as a critical tool due to its advantages in handling multiphysics coupling problems. Existing numerical models have successfully integrated electromagnetic fields, induced currents, heat transfer, and momentum transport into a unified framework [8,9,10]. Nevertheless, these models are generally limited to melting processes involving homogeneous materials, with insufficient consideration given to regions with graded physicochemical properties. Furthermore, current research has predominantly focused on the heating phenomena in solid and liquid states during VIM, while the key process of solid/liquid phase transition in materials with heterogeneous properties has been largely overlooked [11,12,13]. When melting raw materials with non-uniform physical characteristics, the latent heat of phase transition significantly influences the evolution of the temperature field—a core factor that has not been adequately addressed in existing studies.

Extensive research over the past few decades has elucidated the melting behavior of bulk alloy during induction melting, including solid/liquid phase transitions, natural convection, and electromagnetic stirring [9,14,15]. These mechanisms are all involved in practical powder recycling production. However, due to the distinct melting characteristics of powder/bulk composite, their co-melting behavior exhibits significant differences compared to the melting of bulk alloy alone. Therefore, investigating the co-melting mechanisms of powder/bulk composite with heterogeneous melting characteristics is crucial for advancing their large-scale production and industrial applications.

Improving the utilization efficiency of recycled FGH96 alloys constituted a core engineering motivation of this work. To achieve this target, this study elucidated the induction melting characteristics of powder/bulk composite and develops a multiphysics coupling model for the melting process. The heat and mass transfer mechanisms governing the induction co-melting of recycled FGH96 powder bed and bulk alloy are systematically investigated. The key scientific novelty lies in the establishment of a multiphysics model dedicated to powder/bulk composite melting system. This work fills the research gap in the melting behaviors of compositionally identical superalloys with distinct morphologies, providing fundamental scientific insights. Furthermore, the obtained findings provide a rigorous theoretical basis for optimizing powder recycling strategies and improving material utilization efficiency, thereby offering substantial engineering application value.

## 2. Experimental Platform and Mathematical Model

### 2.1. Geometric Model and Assumptions

**Figure 1:**
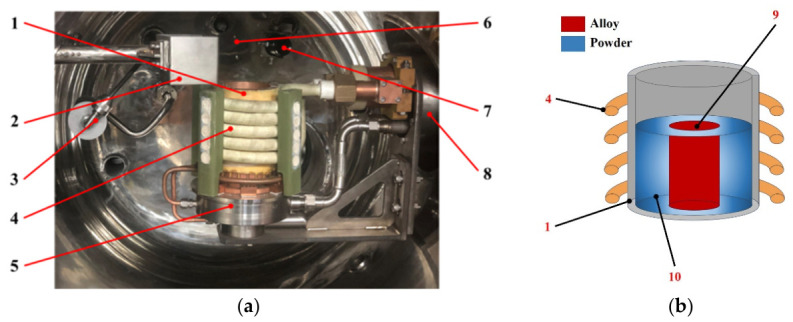
(**a**) Vacuum induction melting system: 1—melting crucible, 2—charging device, 3—rapid immersion thermocouple, 4—water-cooled inductor, 5—coil cooling device, 6—heat shield, 7—online infrared camera, 8—melting crucible tilting device; (**b**) two-dimensional axisymmetric geometric model: 9—alloy, 10—powder.

A VIM induction melting facility developed by the Chinese Academy of Sciences (Beijing, China) was utilized for model calibration and experimental validation, as shown in Figure 1a. The VIM system comprises a vacuum-pumped sealed steel chamber, in which a melting unit with a 500 g nickel-based superalloy capacity is installed. The induction melting assembly is mainly composed of a sinusoidal coil and a magnesium oxide (MgO) crucible. The water-cooled copper coaxial coil is driven by a 50 kW power supply operating at 3 kHz, with an external chiller providing continuous circulating cooling.

The schematic of the computational domain, VIM system, and material layout is displayed in Figure 1b. Given the axial symmetry and periodic structural characteristics of the melting chamber, the numerical model was simplified to a two-dimensional axisymmetric plane. The induction coil is energized with periodically alternating current, and its electrical parameters can be maintained steadily under the fixed power output of the generator. In the numerical setup, the initial state of the alloy workpiece was defined as fully solid. The solid/liquid phase transformation behavior was considered throughout the simulation, while the solidification process was neglected. Furthermore, the high-vacuum operating environment eliminates oxide particle formation on the molten pool surface, which is reasonably assumed in the present model.

In this study, the FEM embedded in COMSOL Multiphysics^®^ (v. 6.1, COMSOL AB, Stockholm, Sweden) was adopted to discretize the partial differential equations that govern multiphysical field behaviors. The electromagnetic field was solved across all computational domains, while the molten region was designated as the computational domain for the fluid dynamic field. Numerical investigations into heat transfer characteristics were performed for all computational regions, including the melting chamber, crucible, induction coil, and alloy materials. Different time scales between electromagnetic induction, heat transfer, and fluid flow necessitate frequency-domain analysis for the electromagnetic field and time-domain calculation for the thermal field and the fluid dynamic field. The detailed governing equations are presented in the next section.

Heat loss calculation in the simulation mainly considers surface radiation. Radiation heat loss on the molten pool surface was computed via the Stefan–Boltzmann law relating surface emissivity to melt temperature. In addition, radiative heat input from the surrounding environment to the melt was ignored. A perfect vacuum condition was assumed inside the chamber, thereby excluding air convection heat transfer effects from the numerical solution.

### 2.2. Mathematical Model

#### 2.2.1. Electromagnetic Sub-Model

According to the Maxwell–Ampère law, the governing equation for the electromagnetic field is formulated as

(1) $$\nabla \times H=j\omega_{f}D+J$$

where *H*, *D*, *J*, and ω*_f_* are the magnetic field strength, electric displacement vector, current density, and angular frequency, respectively.

With consideration of the Lorentz term in Ohm’s law for molten metal flow *J* = *σE* + *σ*(*u* × *B*) + *J*_e_, Equation (1) can be obtained by derivation as [12]

(2) $$\nabla \times H=\sigma E+\sigma \left(u\times B\right){+J}_{e}+j\omega_{f}D$$

where *σ*, *E*, *u*, and *J*_e_ are the electrical conductivity, electric field strength, melt flow velocity vector, and source current density, respectively.

The resulting partial differential equation for the magnetohydrodynamic sub-model is derived by incorporating the magnetic vector potential *B* = ∇ × *A* and *E* = −j*ωA*, together with the constitutive relations *B* = μ_0_μ_r_*H* and *D* = ε_0_ε_r_*E*; Equation (2) can be obtained by derivation as

(3) $$J=\left(j\omega_{f}\sigma -\omega_{f}^{2}\varepsilon_{0}^{-1}\varepsilon_{r}^{-1}\right)A+\nabla \times \left(\mu_{0}^{-1}\mu_{r}^{-1}\nabla \times A\right)-\sigma u\times \left(\nabla \times A\right)+J_{e}$$

where ε_0_, ε_r_, μ_0_, μ_r_, and *A* are the vacuum permittivity, relative permittivity, vacuum magnetic permeability, relative magnetic permeability, and magnetic vector potential, respectively.

#### 2.2.2. Thermal Sub-Model

A secondary electromagnetic effect induced by the alternating magnetic field is inductive heating. This thermal phenomenon originates from eddy current dissipation and is positively correlated with the material electrical conductivity, as well as the coupling effect of electric field intensity and current density [16].

(4) Qe=12ReJ×E*

where *Q_e_* denotes the heat generated by induction heating and is treated as a source term.

According to the Fourier heat conduction equation, the governing equation for thermal field is formulated as [17]

(5) $$\rho C_{p}\left(\frac{\partial T}{\partial t}+\left(u\cdot \nabla T\right)\right)=\nabla \cdot \left(\lambda_{\mathrm{eff}}\nabla T\right)+Q_{e}$$

where ρ, C_p_, and *T* are the fluid density, specific heat capacity, and temperature field, respectively. The factor λ_eff_ is the sum of the contribution of the liquid metal thermal conductivity and turbulent thermal conductivity.

#### 2.2.3. Magnetohydrodynamics Sub-Model

The electromagnetic field produces turbulent circulating stirring on liquid metal flow. The Navier–Stokes equations govern momentum transport. For incompressible Newtonian fluids, transient formulations are derived in accordance with mass and momentum conservation laws [18]:

(6) $$\frac{d\rho }{dt}+\nabla \cdot \left(\rho u\right)=0$$

(7) $$\rho \left(\frac{\partial u}{\partial t}+u\cdot \nabla u\right)=-\nabla p+\nabla \cdot K+F_{L}+F_{B}+S\left(T\right)\cdot u$$

where p, *F*_L_, *F*_B_, K, and S(*T*) are the pressure, the Lorentz force, the buoyancy force, the stress tensor, and an additional source term that contains the Carman–Kozeny relation, respectively.

(8) $$F_{B}=-\rho \cdot \beta \cdot g\cdot \left(T-T_{\mathrm{PC}}\right)$$

where β, g, and *T*_PC_ are the volumetric thermal expansion coefficient, gravitational force, and phase transformation temperature, respectively.

The Reynolds-averaged Navier–Stokes model combined with standard wall function method is adopted to resolve the mean velocity field of turbulent flow and predict the flow velocity within the viscous sublayer. This numerical scheme boasts favorable convergence efficiency and cost-effective calculation performance, thus serving as a reasonable simulation approach. Two extra solving variables, namely, the turbulent kinetic energy (*k*) and turbulent dissipation rate (*ε*), are introduced, with respective transport equations established correspondingly.

The transport equation governing turbulent kinetic energy *k* is formulated as [12]

(9) $$\rho \frac{\partial k}{\partial t}+\rho u\cdot \nabla k=\nabla \left(\left(\mu +\frac{\mu_{T}}{\sigma_{k}}\right)\nabla k\right)+P_{k}-\rho \varepsilon$$

The transport equation governing turbulent dissipation rate *ε* is formulated as

(10) $$\rho \frac{\partial \varepsilon }{\partial t}+\rho u\cdot \nabla \varepsilon =\nabla \left(\left(\mu +\frac{\mu_{T}}{\sigma_{\varepsilon }}\right)\nabla \varepsilon \right)+C_{\varepsilon 1}\frac{\varepsilon }{k}P_{k}-C_{\varepsilon 2}\rho \frac{\varepsilon^{2}}{k}$$

The turbulent production rate is formulated as

(11) $$P_{k}=\mu_{T}\left(\nabla u:\left(\nabla u+\nabla u^{T}\right)\right)$$

The turbulent viscosity is formulated as

(12) $$\mu_{T}=\rho C_{\mu }\frac{k^{2}}{\varepsilon }$$

where *P*_k_, *μ*, and *μ_T_* are the turbulent production rate, dynamic viscosity, and turbulent viscosity, respectively. The model constants *C*_ε1_, *C*_ε2_, *C_μ_*, *σ_k_*, and *σ_ε_* are assigned standard values of 1.44, 1.9, 0.09, 1.0, and 1.3, respectively.

As the fundamental driving source triggering fluid motion within the magnetohydrodynamic coupling system, the Lorentz force endows the fluid medium with kinetic energy while generating radial electromagnetic stress that propels molten fluid to detach from the crucible boundary and migrate toward the interior region. This physical force can be mathematically defined as the cross product formed by the magnetic field and induced current existing inside the conductive fluid medium.

The Lorentz force is formulated as

(13) FL=12ReJ×B*

#### 2.2.4. Phase Sub-Model

The solid/liquid phase transition of superalloy melt was numerically solved via the enthalpy/porosity mushy zone method. The piecewise linear liquid fraction function φ(*T*) was adopted to quantify the volume fraction of liquid alloy within the mushy zone, and the Darcy damping source term derived from the Carman–Kozeny relation was introduced into the momentum equation to describe flow resistance induced by the solid phase.

The piecewise linear liquid fraction function is formulated as [19]

(14) $$\left\{\begin{array}{ll} \varphi \left(T\right)=0 & \;T<T_{\mathrm{PC}}-\frac{\Delta T_{\mathrm{PC}}}{2}\\ \varphi \left(T\right)=\frac{T-\left(T_{\mathrm{PC}}-\frac{\Delta T_{\mathrm{PC}}}{2}\right)}{\Delta T_{\mathrm{PC}}} & \;T_{\mathrm{PC}}-\frac{\Delta T_{\mathrm{PC}}}{2}<T<T_{\mathrm{PC}}+\frac{\Delta T_{\mathrm{PC}}}{2}\\ \varphi \left(T\right)=1 & \;T>T_{\mathrm{PC}}+\frac{\Delta T_{\mathrm{PC}}}{2} \end{array}\right.$$

The source term is formulated as

(15) $$S\left(T\right)=A_{m}\frac{{\left(1-\varphi \left(T\right)\right)}^{2}}{\varphi {\left(T\right)}^{3}+\varepsilon }$$

where Δ*T*_PC_, A_m_, and **ε** are the parameter phase change temperature range, the mushy zone factor, and a constant (**ε** = 0.001 prevents division by zero for the case φ(*T*) = 0), respectively.

### 2.3. Boundary Conditions

In the thermal field simulation, radiative boundary conditions were imposed on the molten pool surface. The numerical calculation considered surface emissivity, the Stefan–Boltzmann constant, and the temperature difference between the molten pool surface and the 25 °C infinite ambient boundary. The outer wall of the melting chamber was defined as a thermally insulated boundary with zero heat flux. These thermal boundaries were combined with the above-mentioned flow and turbulence constraints to guarantee the integrity of the multiphysics computational framework.

### 2.4. Mesh Generation and Solver Scheme

**Figure 2:**
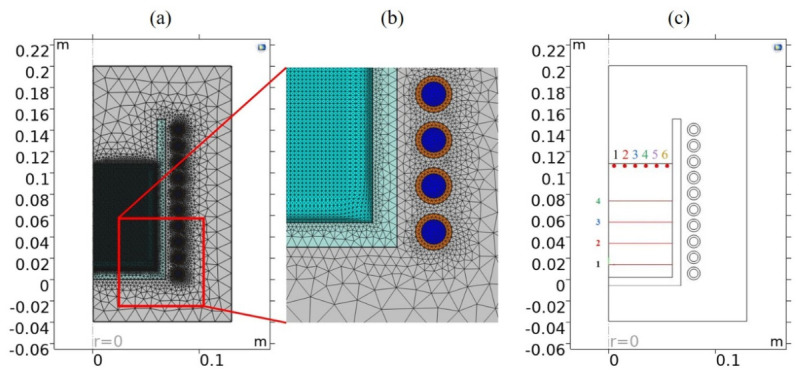
Geometric models and meshes in computational domains: (**a**) grid diagram; (**b**) partial grid diagram; (**c**) data collection points and lines.

High-quality mesh discretization is critical for the numerical accuracy of coupled nonlinear fluid and electromagnetic fields. A two-stage meshing strategy was adopted for different simulation purposes. Unstructured triangular meshes with optimized minimum element sizes were applied in mesh deformation calculations to accurately capture the electromagnetic skin effect. Boundary layer meshes were generated for the coil domain to resolve resistive losses caused by coil self-inductance, while ultra-fine triangular meshes were assigned to other computational domains, as shown in Figure 2a. The data sampling positions for follow-up studies are indicated in Figure 2b.

The flow and thermal fields were discretized with second-order accuracy, while quadratic finite elements were adopted to solve for the magnetic vector potential for high-precision electromagnetic field calculation. A segregated solver was applied for multiphysics iteration. This solver decouples the coupled physical problem and solves individual modules sequentially in each iteration. The time step was set to 0.02 s for free surface simulation. All physical fields were computed using the built-in direct solvers in COMSOL Multiphysics^®^ (v. 6.1, COMSOL AB, Stockholm, Sweden).

### 2.5. Material Properties

The FGH96 bulk alloy and FGH96 powder adopted for the verification experiments in this study were supplied by the Chinese Academy of Sciences (Beijing, China). The thermophysical and electromagnetic parameters required for solving the above governing equations are summarized in Table 1. In the electromagnetic calculation, all materials are defined as paramagnetic media with a relative permeability of μ_r_ = 1, and the crucible and melting chamber are set as electrical insulators with a conductivity of σ = 0. The thermal and electrical parameters of the copper induction coil are derived from the built-in material database of COMSOL Multiphysics^®^ (v. 6.1, COMSOL AB, Stockholm, Sweden). The loose powder bed has a solid volume fraction of 53%, and the corresponding effective density of the powder domain is calculated by the volume-weighted homogenization method based on the intrinsic density of the fully dense FGH96 superalloy. In the same way, the effective specific heat capacity of the powder bed is obtained through volume averaging of solid alloy and vacuum voids. The abundant vacuum interstices between particles significantly degrade heat conduction performance [20]. A low effective thermal conductivity is reasonably assigned to the powder domain with reference to a published study [20] to characterize the authentic heat transfer behavior of loosely packed powder bed.

### 2.6. Model Calculation and Coupling

In this work, COMSOL Multiphysics^®^ (v. 6.1, COMSOL AB, Stockholm, Sweden) with powerful pre- and post-processing functions was adopted to realize the numerical calculation and multi-field coupling of each sub-model. The unified simulation platform guarantees high computational accuracy and effectively compensates for the deficiencies of separate modeling methods adopted in previous studies. To facilitate the iterative solution of the multiphysics system, the electromagnetic sub-model was solved firstly using a frequency-domain solver, which outputted the electromagnetic force and Joule heat source terms required for subsequent calculation. Thereafter, the obtained force source data were imported into the multiphase flow module to couple with the fluid dynamic model, thereby achieving the synchronous solution of flow field characteristics.

### 2.7. Experimental Procedures

In situ full-field temperature measurements were performed to provide baseline data for subsequent comparative analysis and model validation. The transient temperature response of molten alloy under varying power conditions was analyzed to verify the accuracy of temperature field coupling. An Optris PI 400i infrared camera (Optris GmbH & Co. KG, Berlin, Germany) mounted inside the vacuum chamber right above the horizontal plane of the crucible was utilized for in situ temperature monitoring, with a measurable upper limit of 1500 °C and a measurement accuracy of 2%. All infrared temperature measurements were conducted during the melting stage of the charge to guarantee the validity of the experimental results. Owing to the limitations of the experimental conditions, the maximum material size used for validation experiments in the present study is 100 mm, and the numerical simulation scheme is revised on the basis of measured data obtained from practical validation tests. Although this method can fully capture the temperature variations associated with phase transformation on the upper surface of the material, direct experimental evidence for the temperature evolution within the central region of the melt pool remains insufficient.

## 3. Results and Discussion

### 3.1. Magnetic Field

**Figure 3:**
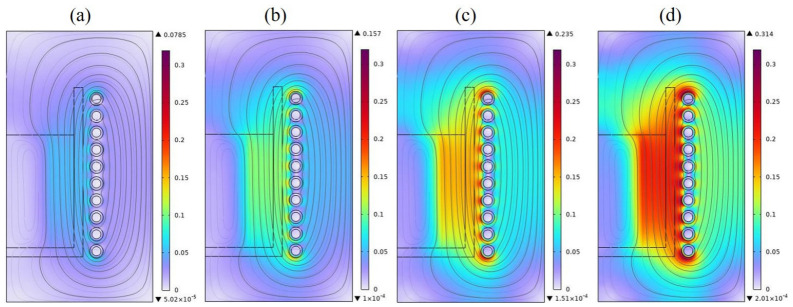
Distribution of magnetic induction at different coil currents: (**a**) 1000 A; (**b**) 2000 A; (**c**) 3000 A; (**d**) 4000 A.

**Figure 4:**
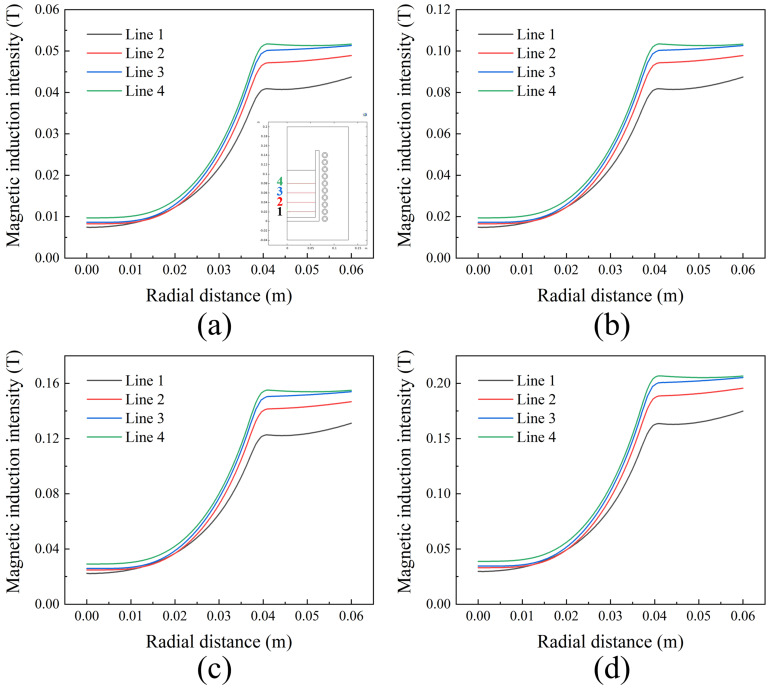
Numerical results of magnetic induction at different coil currents: (**a**) 1000 A; (**b**) 2000 A; (**c**) 3000 A; (**d**) 4000 A.

The distribution features of magnetic induction intensity inside the initial molten pool of FGH96 powder/bulk composite under different coil currents are shown in Figure 3. The simulation results show that as the coil current increases proportionally, the penetration of the magnetic field generated by the induction coil is improved, leading to higher magnetic induction intensity across most regions of the molten pool. With the gradual increase in current magnitude, the high-strength magnetic field area (red region) at the melt edge expands continuously. Meanwhile, an enhanced magnetic field zone (blue region) appears within the melt interior and progressively expands in coverage. This distribution feature is dominated by the electromagnetic skin effect. Owing to the magnetic shielding effect of the molten alloy, the radial magnetic field intensity decays gradually from the melt boundary to the inner region, as shown in Figure 4a–d. In comparison with the conventional VIM of bulk alloy, the powder accumulation region presents weaker electromagnetic shielding capability, which, however, exerts no negative impact on the overall melting behavior of the FGH96 superalloy.

### 3.2. Fluid Flow Field

#### 3.2.1. Evolution of Flow Field

**Figure 5:**
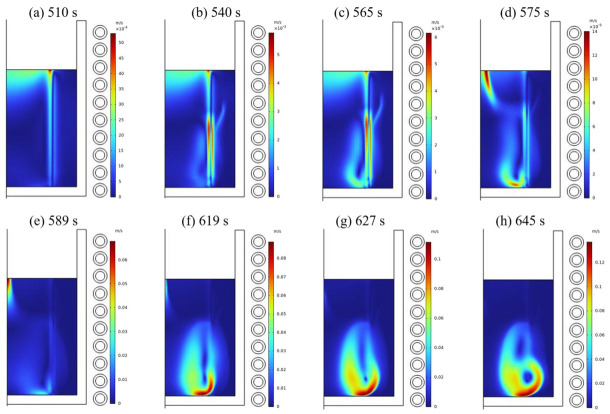
Flow field at different time steps: (**a**–**h**) different steps.

A transient current pre-simulation is implemented to acquire precise initial flow field distribution and characterize the transient evolutionary behavior of fluid flow. Electromagnetic force acts as the dominant driving factor for fluid motion in the computational domain. The correlation between current magnitude and flow dynamic characteristics can be intuitively reflected through the variation in melt flow velocity. Figure 5 presents the flow field simulation results of FGH96 powder/bulk composite melt during VIM. Figure 5a,b show that the tendency of flow starts at the top edge of the bulk alloy. As the VIM continues, the flow region shifts toward the bottom of the bulk alloy (Figure 5c,d), and the flow begins at the bottom of the bulk alloy, which means that the melting starts in this region first (Figure 5e,f). Along with the progress of VIM, the molten pool enters a flow state and invades the powder. Due to the combined coupling effect of electromagnetic stirring force and gravity, the vortex region gradually forms inside the melt, and the complex flow gradually forms an enormous cyclic vortex (Figure 5g,h). This facilitates the uniform flow inside the melt, resulting in reduced segregation of the melt.

#### 3.2.2. Current Dependence of Flow Field

**Figure 6:**
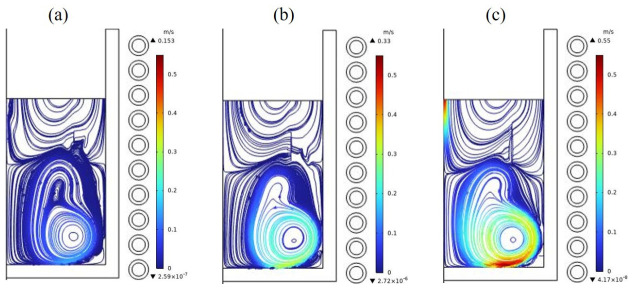
Flow field at different coil currents: (**a**) 1000 A; (**b**) 2000 A; (**c**) 3000 A.

Analysis of the streamlines in Figure 6 shows that vortex zones are uniformly distributed throughout the melt at various coil currents. These zones tend to occur in regions of nearly equal velocity. As presented in Figure 6a, the melt outside the vortex zones possesses a higher velocity gradient, whereas the velocity variation inside is insignificant. The position of vortex zones hardly changes with increasing coil current, indicating that current variation exerts little influence on the overall flow direction. Nevertheless, as the current rises, the flow velocity increases across all regions, streamlines become smoother, and the number of vortex zones drops slightly. As shown in Figure 6c, the number of vortex zones reaches its minimum at a coil current of 3000 A. The morphology of small-scale vortices is insensitive to current variations. Vortex zones near the melt surface frequently trigger liquid level fluctuations, which intensify as the current rises. Such fluctuations result in surface splashing and air entrainment, compromising the metallurgical quality.

**Figure 7:**
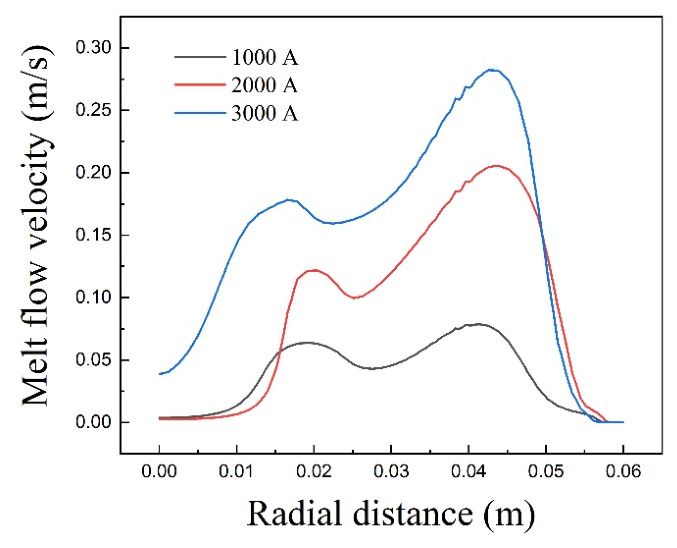
Numerical results of melt flow velocity at different coil currents.

Figure 7 illustrates the impact of varying coil currents on the flow field. At a coil current of 1000 A, the velocity at the melt core is exceptionally low. The highest velocity is observed near the boundaries of the bulk alloy and powder. With increasing coil current, the melt velocity increases proportionally, and the red high-velocity zone spreads from the bulk alloy boundaries to the powder region. Simultaneously, the blue region, representing low velocity, diminishes. Thus, increasing the coil current causes a higher melt velocity, which, in turn, enhances the melting efficiency. This implies that a higher current leads to improved melting performance.

### 3.3. Temperature Field

#### 3.3.1. Evolution of Temperature Field

**Figure 8:**
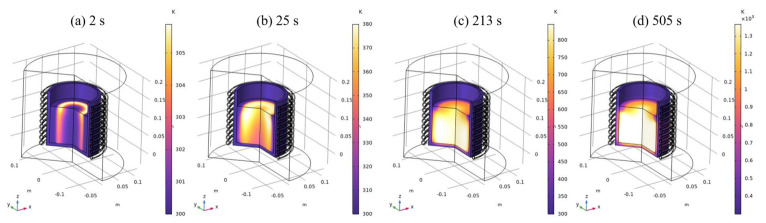
Evolution of temperature field at different time steps: (**a**–**d**) different steps.

A ten-minute frequency-transient simulation was performed to acquire the temperature field of powder/bulk composite during pre-melting heating in induction melting, and to characterize the transient evolution of temperature distribution as the molten pool develops. This simulation aims to reproduce the distinctive heating characteristics and solid/liquid phase transition in the combined induction melting of powder/bulk composite. Figure 8 illustrates the simulated temperature field of the FGH96 alloy melt during the VIM process. With increasing coil current, the high-temperature region within the melt, represented by the yellow-colored area, contracts slightly inward in the horizontal direction while extending downward in the vertical direction (Figure 8a). This behavior indicates that the heating in VIM initiates at the boundary of the bulk alloy and progresses inward. The skin effect inherent to vacuum induction heating causes the bulk alloy to heat and melt before the surrounding powder (Figure 8b). Once the bottom portion of the bulk alloy transitions to liquid, the melt begins to infiltrate the powder, which subsequently melts as well (Figure 8c,d). With the growing volume of melt inside the crucible, the powder softens and agglomerates progressively, speeding up melting. The transient current analysis was conducted to determine the precise initial temperature distribution and model the transient variation in the temperature field. The present simulation captures the heating behaviors in the combined induction melting of powder/bulk composite. The temperature field magnitude and distribution are fully considered, playing a vital role in revealing the melting mechanism of the mixed powder/bulk composite system.

#### 3.3.2. Current Dependence of Temperature Field

**Figure 9:**
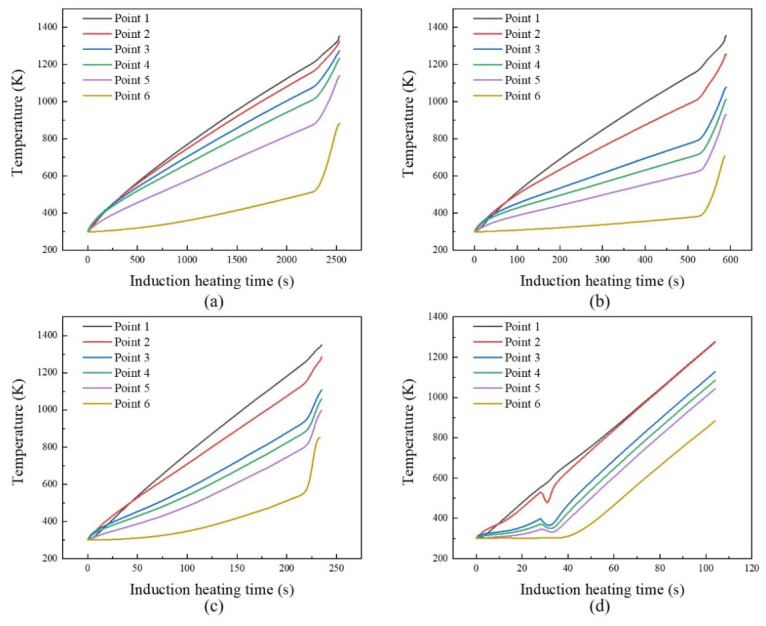
Numerical results of melt temperature at different coil currents: (**a**) 1000 A; (**b**) 2000 A; (**c**) 3000 A; (**d**) 4000 A.

Figure 9 illustrates the effects of coil currents on the temperature field of the melt. Due to the approximate axial symmetry of the melt temperature field, the radial temperature distribution was investigated on the horizontal cross-section under different current parameters. The simulation results reveal a prominent radial temperature gradient inside the melt, which expands continuously with the increase in coil current. This variation mechanism can be explained by the enhanced induced current in the bulk alloy region under elevated coil current, which significantly improves the induction heating efficiency. The temperature rise of the powder region mainly relies on thermal conduction from the bulk alloy matrix. The temperature growth rate of the bulk alloy region driven by intensified induction heating is far higher than that of the powder region relying on interfacial heat conduction. Moreover, the powder region adjacent to the low-temperature crucible wall suffers partial heat dissipation. Accordingly, the powder region near the crucible edge exhibits a much lower heating rate compared with the bulk alloy region, and this temperature discrepancy is further aggravated with rising coil current, ultimately enlarging the radial temperature difference of the melt.

### 3.4. Phase Field

**Figure 10:**
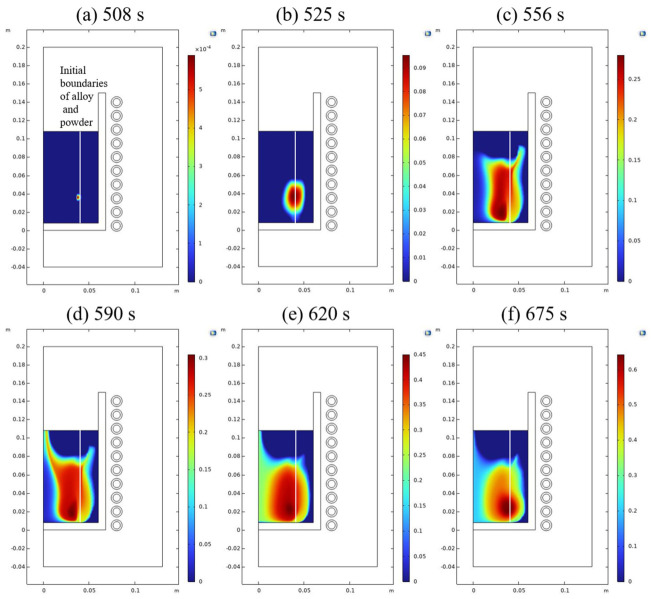
Evolution of phase field at different time steps: (**a**–**f**) different steps.

The formation of the molten pool is influenced by the temperature field, initiating in the regions where the powder/bulk composite first reaches its melting point. Once the molten pool is formed, it begins to expand under the combined effects of natural convection and electromagnetic stirring, as the temperature of the base alloy continues to rise. With the ongoing melting of powder/bulk composite, the molten pool gradually expands. Figure 10 shows the evolution of the phase field within the powder/bulk composite during VIM. Figure 10a shows that solid/liquid phase transition occurs at the bottom of the superalloy, which corresponds to the temperature and flow fields, and is consistent with the experimental observations. In the VIM process, the initial molten pool of conventional bulk alloy forms at the edge of the alloy, specifically at the boundary of the material region. In contrast, during the co-melting process of powder/bulk composite, the molten pool forms at the interface between the bulk alloy and the powder, typically positioned at the center of the material region (Figure 10b–d). The aforementioned simulation results provide valuable insights into the evolution of the molten pool in the VIM process for materials with different melting behaviors. This understanding is essential for optimizing the design of the melting process, including considerations for material stacking configurations and coil current settings. Ultimately, under the action of gravity, the co-melted molten pool settles to the crucible floor (Figure 10e,f).

### 3.5. Experimental Validation

**Figure 11:**
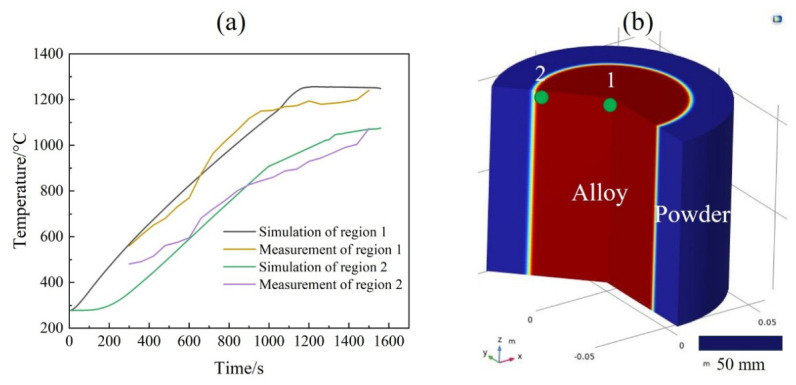
Experimental measurements and simulation results of the temperature field: (**a**) fitted curve; (**b**) data collection points.

Temperature and heat serve as the critical coupling bridge and state-governing variable throughout the fully coupled system encompassing electromagnetic, thermal, magnetohydrodynamic, and solid/liquid phase fields. Therefore, accurate temperature prediction fundamentally validates the reliability of the present multiphysics numerical model. On this basis, experimental melt temperature measurements were performed to quantitatively verify the computational accuracy and physical rationality of the aforementioned multiphysics coupling framework integrating electromagnetic response, heat and mass transfer, magnetohydrodynamic flow, and solid/liquid phase transition. To comprehensively validate the model’s capability in characterizing the unique melting behaviors of bulk alloy matrix and powder/bulk composite system, two typical monitoring positions were arranged, including the center of bulk alloy (region 1) and the interfacial zone between powder and bulk alloy (region 2). The horizontal distribution of monitoring points in the molten pool is displayed in Figure 11b. In region 1, the Pearson correlation coefficient between experimental measurements and numerical predictions of temperature evolution is 0.9781 (*p* < 0.0001), presenting excellent linear consistency. The perfect synchronization of experimental and simulated time axes with a correlation coefficient of *r* = 1 eliminates analytical errors induced by time mismatch in transient multi-field coupling calculations. In region 2, dominated by intense electromagnetic-thermo-hydrodynamic coupling during powder/bulk co-melting, the temperature correlation coefficient reaches 0.99454 (*p* < 0.0001), with highly consistent temporal variation trends and fully synchronized time axes (*r* = 1). The higher prediction accuracy for the composite melting interface further indicates that the established multiphysics coupling algorithm can accurately resolve the intrinsic heat and mass transfer mechanisms of the powder/bulk composite system. The well-matched temperature results between simulation and experiment at different characteristic regions demonstrate that the proposed model can faithfully reproduce the transient VIM behaviors of recycled FGH96 powder/bulk composite. This fully verifies the rationality of the model governing equations, physical parameter settings, confirming that the developed numerical framework possesses high computational precision and robust applicability for powder/bulk composite melting simulations.

## 4. Conclusions

A 2D coupled numerical model integrating electromagnetic field, flow field, and phase field was established. By combining numerical simulation with experimental validation, we investigated the melting behavior of powder bed/bulk alloy under the skin effect during vacuum induction melting, together with the melt flow characteristics after co-melting of the mixed materials. The conclusions are summarized as follows:

(1) This model offers theoretical support for designing superalloy powder recycling processes, which helps enhance material utilization and lower production costs.
(2) The present model accurately predicts the skin effect evolution and temperature field distribution characteristics. Experimental verification confirms the reliability of the model, laying a foundation for subsequent process parameter optimization.
(3) During VIM of powder bed/bulk alloy, the initial molten pool preferentially forms at the powder/bulk interface, which is distinctly different from the VIM behavior of single bulk superalloy.
(4) Increasing the current reduces the number of vortex zones in the melt. The generated vortices promote sufficient infiltration of the melt into the powder layer and further homogenize the temperature field distribution.

## Figures and Tables

**Table 1:** Material properties for the implemented domains [21,22,23].

Material	Density ρ (kg·m^−3^)	Thermal Conductivity λ (W·m^−1^·K^−1^)	Heat Capacity C_p_ (J·kg^−1^·K^−1^)	Electrical Conductivity σ (S·m^−1^)	Dynamic Viscosity μ (kg·m^−1^·s^−1^)
FGH96	8300	30	400	1	5.4 × 10^−3^
FGH96 powder	4400	0.1	212	0	5.4 × 10^−3^

## Data Availability

The original contributions presented in the study are included in the article, further inquiries can be directed to the corresponding author.
